# Prion infection impairs lysosomal degradation capacity by interfering with rab7 membrane attachment in neuronal cells

**DOI:** 10.1038/srep21658

**Published:** 2016-02-11

**Authors:** Su Yeon Shim, Srinivasarao Karri, Sampson Law, Hermann M. Schatzl, Sabine Gilch

**Affiliations:** 1 Dept. of Ecosystem and Public Health, Faculty of Veterinary Medicine, University of Calgary, Calgary, Canada; 2 Dept. of Comparative Biology and Experimental Medicine, Faculty of Veterinary Medicine, University of Calgary, Calgary, Canada; 3 Hotchkiss Brain Institute, Cumming School of Medicine, University of Calgary, Calgary, Canada

## Abstract

Prions are proteinaceous infectious particles which cause fatal neurodegenerative disorders in humans and animals. They consist of a mostly β-sheeted aggregated isoform (PrP^Sc^) of the cellular prion protein (PrP^c^). Prions replicate autocatalytically in neurons and other cell types by inducing conformational conversion of PrP^c^ into PrP^Sc^. Within neurons, PrP^Sc^ accumulates at the plasma membrane and in vesicles of the endocytic pathway. To better understand the mechanisms underlying neuronal dysfunction and death it is critical to know the impact of PrP^Sc^ accumulation on cellular pathways. We have investigated the effects of prion infection on endo-lysosomal transport. Our study demonstrates that prion infection interferes with rab7 membrane association. Consequently, lysosomal maturation and degradation are impaired. Our findings indicate a mechanism induced by prion infection that supports stable prion replication. We suggest modulation of endo-lysosomal vesicle trafficking and enhancement of lysosomal maturation as novel targets for the treatment of prion diseases.

Prions are a class of infectious agents that are composed exclusively of protein, namely a misfolded isoform of the cellular prion protein PrP^c^ which is called PrP^Sc ^1,2. Prions replicate autocatalytically by transmitting their aberrant conformation to PrP^c^. Upon direct interaction between PrP^c^ and PrP^Sc^, new PrP^Sc^ molecules are generated. In contrast to PrP^c^, PrP^Sc^ has a high β-sheet content, and forms aggregates and amyloid fibres^3^,^4^. Upon infection of animal or human hosts, PrP^Sc^ aggregates accumulate in the brain of individuals and cause fatal neurodegenerative disorders, e.g. Creutzfeldt-Jakob disease (CJD) in humans, scrapie in sheep, bovine spongiform encephalopathy (BSE) in cattle, or chronic wasting disease in cervids^5^,^6^,^7^.

On a cellular level, PrP^c^ is a plasma membrane protein attached to the cell surface by a glycosylphosphatidyl-inositol (GPI) anchor^8^. In prion-infected cells a portion of PrP^c^ is constantly converted into PrP^Sc^, for which PrP^c^ expression^9^ and its cell surface localisation^10^,^11^,^12^,^13^ are required. Consequently, PrP^Sc^ can be found at the plasma membrane and along the endocytic pathway^14^,^15^. It has been demonstrated that the plasma membrane, recycling endosomes, and multivesicular bodies are cellular compartments of prion conversion^16^,^17^,^18^,^19^,^20^. Whereas it has been shown that prion infection interferes with post-Golgi protein trafficking both in prion infected neuroblastoma (N2a) cells and mouse models of prion infection and thereby suppresses insulin receptor signaling^21^, only little is known about how the accumulation of PrP^Sc^ in endocytic vesicles affects their subcellular trafficking.

Rab proteins comprise a large family of small GTPases which are localized to distinct intracellular membranes and regulate vesicle trafficking. They switch between an inactive, GDP-bound cytosolic state and an active, GTP-bound membrane associated state. Rab proteins are activated by the exchange of GDP with GTP which is catalysed by guanine nucleotide exchange factor (GEF), and membrane association is mediated by C-terminal prenylation. The active form interacts with effector proteins and is inactivated by GTP hydrolysis. Inactive, GDP-bound rabs are recognized and bound to rab GDP dissociation inhibitor (rabGDI) in the cytosol. Membrane properties such as the lipid composition can influence the recruitment of rab proteins to certain compartments^22^,^23^.

Studies on axonal transport in motor neurons of prion infected mice revealed an impairment of retrograde transport, which involves a rab7-mediated pathway^24^. In cells overexpressing mutant PrP the level of functional rab11 was reduced due to an overexpression of RabGDIα, resulting in mutant PrP accumulation in the secretory pathway^25^. In brains from CJD patients enlargement of rab5 and rab7 positive vesicles corresponding to early and late endosomes, respectively, is found associated with PrP^Sc^ depositions^26^. Another study demonstrated that rab7a interacts directly with PrP^c^^27^.

These findings and the described distribution of PrP^Sc^ aggregates in vesicles along the endocytic pathway led us to investigate a possible influence of prion infection on endocytic vesicle trafficking in neuronal cell lines (CAD5 and N2a) persistently infected with prion strain RML and/or 22L.

We found that the amount of membrane-bound rab7 is reduced upon prion infection. Since functional rab7 is involved in lysosomal maturation, we compared the efficiency of lysosomal degradation in N2a and 22LN2a cells. We found a significantly lower degradation rate in prion infected cells. Our data suggest a mechanism induced by PrP^Sc^ accumulation that can support the persistent replication of prions by preventing PrP^Sc^ degradation in lysosomes. Notably, perturbations of the lysosomal degradation pathway may be linked to neurodegeneration and neuronal death, as observed in prion infected individuals. Based on our results, we suggest induction of lysosomal maturation as a target for treatment of prion diseases.

## Results

### Prion infection interferes with rab7 membrane attachment

In prion infected neuronal cells PrP^Sc^ aggregates are mainly located at the cell surface and in vesicles along the endocytic pathway^14^,^15^,^17^,^19^,^28^. We hypothesize that the presence of membrane associated protein aggregates can interfere with vesicle trafficking, and decided to compare the amounts of active, i.e. membrane-bound rab proteins in non-infected and prion-infected CAD5 and N2a cells, respectively. N2a cells were persistently infected with prion strain 22L, whereas CAD5 cells were infected with either 22L or RML prions. We compared levels of rab7 which is a late endosomal rab protein, rab9 which mediates vesicle shuttling between the trans-Golgi network (TGN) and late endosomes, and rab11 which localizes to recycling endosomes^23^.

**Figure 1:**
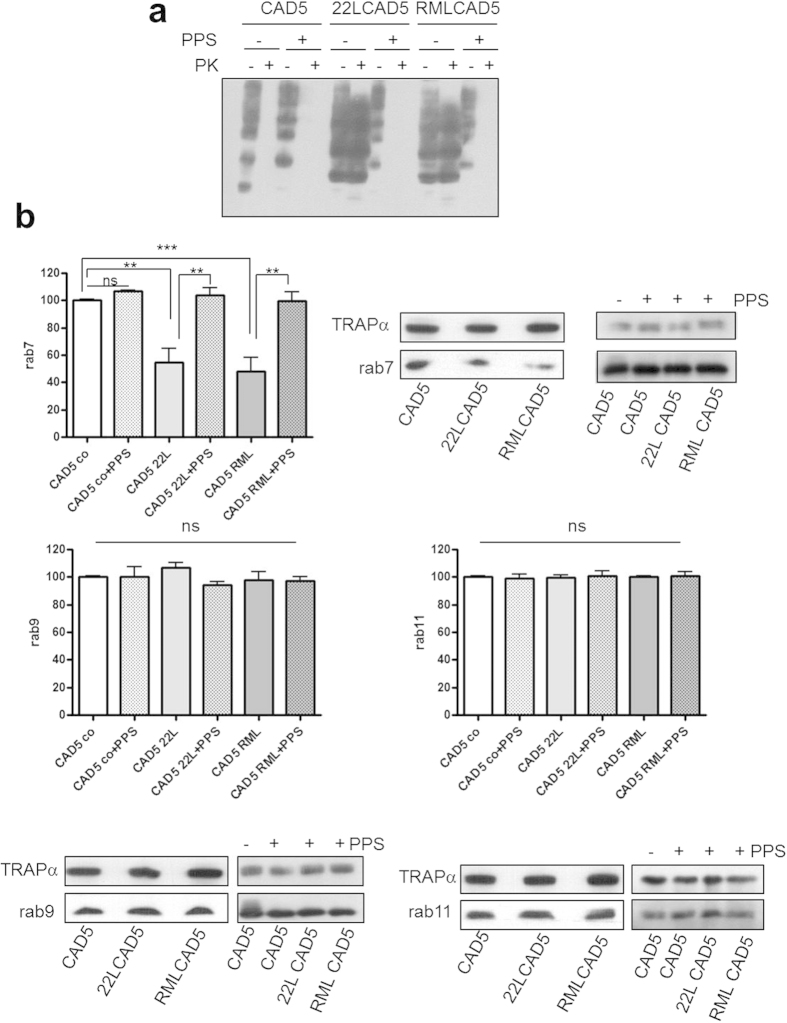
Reduced membrane association of rab7 in CAD5 cells infected with prion strains 22L or RML. (**a**) Lysates of CAD5, 22LCAD5 or RMLCAD5 cells −/+ PPS treatment (1 μg/ml for 10 days) as indicated were subjected to PK digestion (20 μg/ml, 30 min, 37 °C) or not. Aliquots were analysed by immunoblot using monoclonal anti-PrP antibody 4H11. (**b**) Crude membrane preparations (100 μg protein) of CAD5, 22LCAD5 and RMLCAD5 cells −/+PPS as indicated were subjected to SDS-PAGE and levels of rab7, rab9 and rab11, respectively, were analysed by immunoblot. TRAPα served as a loading control. Signals of three independent experiments were quantified by ImageQuant TL (GE Healthcare) and statistical evaluation using one-way ANOVA test, followed by post-hoc analysis with Tukey’s test (GraphPad Prism software; **p-value < 0.01; ***p-value < 0,001; ns = not significant). Bars represent standard deviation.

**Figure 2:**
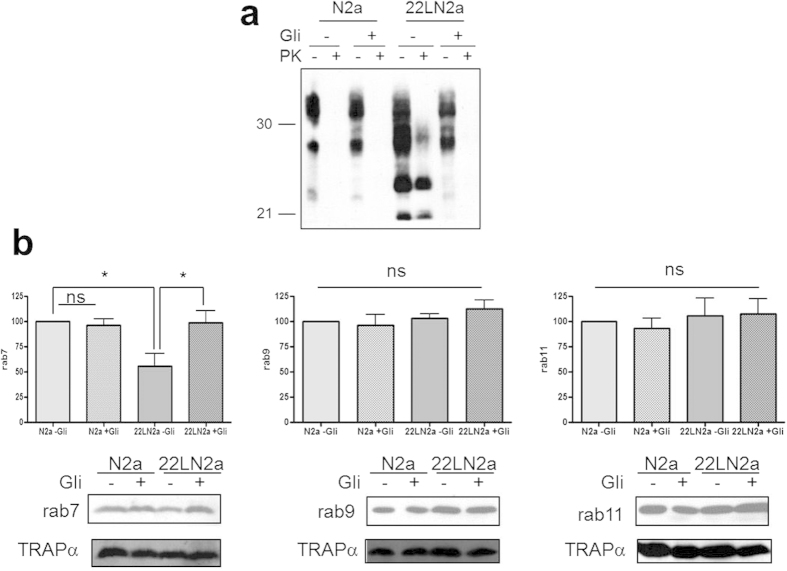
Levels of membrane-bound rab7 are reduced in 22LN2a cells. (**a**) N2a and 22LN2a cells were treated for 10 days with glivec (+ Gli; 10 μM) or not (−Gli). Cells were lysed and lysates were subjected to PK digestion (+PK; 20 μg/ml) or not (−PK). Aliquots were analysed by immunoblot using anti-PrP mAb 4H11. (**b**) Crude membrane extracts (100 μg protein) of N2a or 22LN2a cells treated with glivec (+Gli) or not (−Gli) were subjected to immunoblot and analysed for rab7, rab9 or rab11 levels. TRAPα served as a loading control. Signals of three (+Gli) or six (−Gli) independent experiments were densitometrically quantified and statistically evaluated using one-way ANOVA and Tukey’s test (GraphPad Prism; *p-value < 0.05; ns = not significant). Bars represent standard deviation.

We prepared crude membrane fractions of CAD5, 22LCAD5, RMLCAD5 (Fig. 1), N2a and 22LN2a (Fig. 2) cells. As a control, we used homologous cells cured of prion infections by treatment with pentosan polysulfate (PPS), a substance known to interfere with PrP^Sc^ propagation^29^, or with glivec, which increases the degradation rate of PrP^Sc ^30. In order to determine if cells were cured successfully, we subjected lysates of CAD5, 22LCAD5, RMLCAD5 and their PPS treated counterparts to proteinase K (PK) digestion or not and analysed aliquots by immunoblot using the monoclonal anti-PrP antibody 4H11. Whereas in the non-treated 22LCAD5 and RMLCAD5 high amounts of PK resistant PrP^Sc^ were detectable, in both non-infected and PPS treated CAD5 cells no PrP^Sc^ was found (Fig. 1a), demonstrating that CAD5 cells were cured from prion infection at least to an extent that PrP^Sc^ levels were below the detection limit of immunoblots. Next, we analysed the amounts of rab7, rab9 and rab11, respectively, contained in the crude membrane preparations by immunoblot. Equal amounts of protein were loaded, in addition the ER membrane protein TRAPα served as a loading control. Whereas rab9 and rab11 levels did not differ between CAD5, 22LCAD5 and RMLCAD5 cells with or without PPS treatment, a statistically significant reduction of rab7 to approximately 50% of the non-infected cells was observed in 22LCAD5 and RMLCAD5 (Fig. 1b). However, in 22LCAD5 and RMLCAD5 cured with PPS, the rab7 signals were comparable to those in non-infected CAD5 cells and significantly higher than those found in their non-treated counterparts. Of note, PPS treatment did not affect the levels of any of the tested rab proteins in non-infected cells.

**Figure 3:**
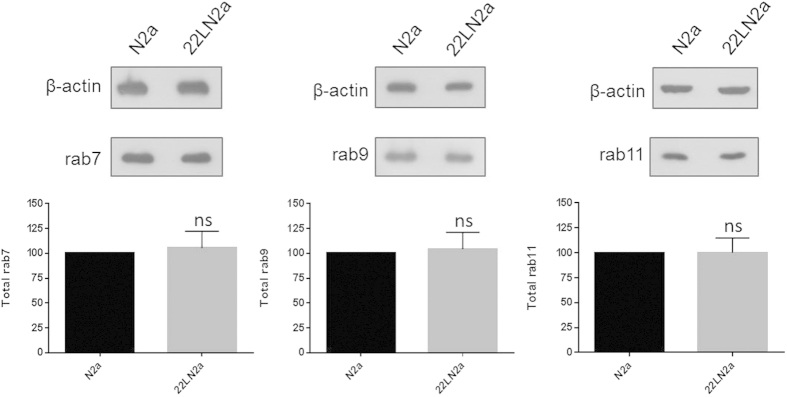
No effect on total levels of rab7 in 22LN2a cells. Overall levels of rab7, rab9 and rab11, respectively, in cell lysates of N2a and 22LN2a cells. β-actin served as a loading control. Average values of 3 independent experiments are shown; statistical analysis was performed using student’s t-test (ns = not significant; GraphPad Prism software). Bars represent standard deviation.

In order to confirm that the observed reduction of membrane-bound rab7 was not specific for one cell line, we performed a similar analysis using N2a and 22LN2a cells. 22LN2a cells were cured from prion infection by treatment for 10 days with the drug glivec (STI571) which induces autophagy^31^ and thereby increases degradation of PrP^Sc ^30. After omitting the drug the amounts of PrP^Sc^ in treated (+Gli) and untreated (−Gli) N2a and 22LN2a cells upon PK digestion of cell lysates were analysed to confirm the absence of PrP^Sc^ (Fig. 2a). Whereas non-treated 22LN2a cells (Fig. 2a) as well as crude membrane preparations of 22LN2a cells (Fig. S1) contained PrP^Sc^, 22LN2a + Gli cells treated with glivec as well as N2a cells (−/+Gli) and membrane preparations of these cells did not harbor detectable levels of PK-resistant PrP (Fig. 2a and Fig. S1). Next, we used crude membrane preparations of N2a and 22LN2a cells treated or not with glivec and tested for amounts of rab7, rab9 and rab11 by immunoblot. Figure 2b depicts representative immunoblots and quantitative results of membranes prepared from three consecutive passages of cells of glivec treated cells, and six individual experiments for N2a and 22LN2a, respectively, without glivec treatment. In non-treated 22LN2a cells a significant reduction of rab7 levels was observed, similar as in 22LCAD5 and RMLCAD5 cells. In 22LN2a cells cured from prion infection (22LN2a + Gli) this reduction was no longer evident, rab7 levels were significantly higher than in 22LN2a-Gli and comparable to those found in N2a cells. Similar to PPS treatment, also glivec treatment did not significantly influence the amounts of either of the analysed rab proteins in N2a cells (compare N2a −/+Gli). Furthermore, rab9 and rab11 signals were not significantly affected by prion infection (N2a vs. 22LN2a cells without glivec). However, the levels of total, i.e. membrane-bound and cytosolic, rab7 did not significantly differ between N2a and 22LN2a cells (Fig. 3).

In summary, with this set of experiments we were able to demonstrate that crude membrane preparations of prion-infected cells contain less rab7 than those of non-infected cells or cells cured from prion infection. This effect was not specific for the cell line or prion strain used for infection, as all prion-infected cell lines, i.e. 22LCAD5, RMLCAD5 and 22LN2a showed a reduction of membrane-bound rab7.

### Sensitivity to RabGDI extraction is not altered by prion infection

We and others have shown previously that prion infection results in up-regulation of cholesterogenic genes, concomitant with increased levels of unesterified cholesterol both *in vitro* and *in vivo*^32^,^33^,^34^,^35^, which is usually found incorporated into cellular membranes^36^. High membrane cholesterol levels can modulate the tightness of rab protein membrane binding^37^,^38^,^39^. With 22LCAD5, RMLCAD5 and 22LN2a cells harboring diminished levels of membrane-bound rab7, we decided to analyse the extractability of rab 7, 9 and 11 from cellular membranes of N2a and 22LN2a cells in order to determine whether an increased sensitivity to rabGDI extraction accounts for this reduction.

**Figure 4:**
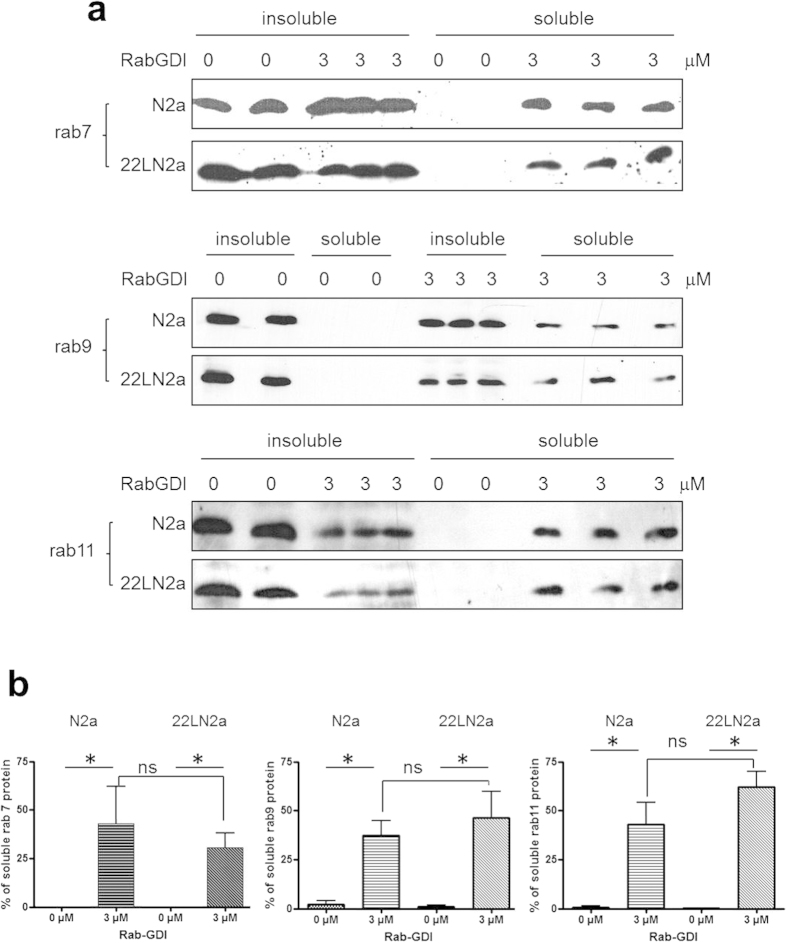
Sensitivity to RabGDI extraction is not disturbed in prion-infected cells. (**a**) Crude membranes of N2a and 22LN2a cells were incubated with 0 (in duplicate) or 3 μM (in triplicate) rab-GDI for 1 hour at 37 °C under constant shaking. Insoluble membrane fractions were separated from soluble fractions by centrifugation (100,000 × g; 10 min). Equal amounts of proteins from soluble and insoluble fractions were subjected to immunoblot analysis using anti-rab7, rab9 or rab11 antibodies. (**b**) Signals of soluble fractions were densitometrically evaluated and expressed as percentage of the total amount of the respective rab protein found in membrane preparations. Triplicate or duplicate values from one experiment were averaged. The averages of at least three independent experiments were statistically analysed using one-way ANOVA and post-hoc analysis with Tukey’s test (*p-value < 0.05; ns = not significant). Bars represent standard deviation.

Therefore, we incubated crude membrane preparations from N2a and 22LN2a cells with 0 or 3 μM of recombinantly expressed and purified rabGDI which solubilises membrane-bound rab proteins^40^. The insoluble membranes and soluble fractions were separated by centrifugation and aliquots of both fractions were analysed by immunoblot using anti-rab7, –rab9 and –rab11, respectively (Fig. 4a). The signals found in soluble fractions upon incubation with 0 or 3 μM rabGDI were quantified. Without rabGDI, none of the rab proteins was detectable in the soluble fraction. However, upon addition of rabGDI, the amount of solubilised rab proteins detected in the supernatant was significantly increased, confirming activity of rabGDI. At least three independent experiments were performed each in triplicate, the average values of the triplicates were used for statistical evaluation. The total amounts of the respective rab protein detected without rabGDI treatment served as 100%. The amounts of solubilised rab proteins were compared between N2a and 22LN2a cells. However, using these conditions, no statistically significant differences were found (Fig. 4b).

Altogether, these data indicate that the strength of rab7, rab9 and rab11 membrane association is not altered upon prion infection.

### Decelerated lysosomal degradation and reduced lysosomal acidification in prion infected cells

Our experiments demonstrate that prion infection results in reduced levels of membrane bound rab7 in persistently prion infected neuronal cells. Association of rab7 with late endosomal membranes is critical for lysosomal maturation, and diminished rab7 expression levels can interfere with lysosomal degradation^41^,^42^,^43^.

**Figure 5:**
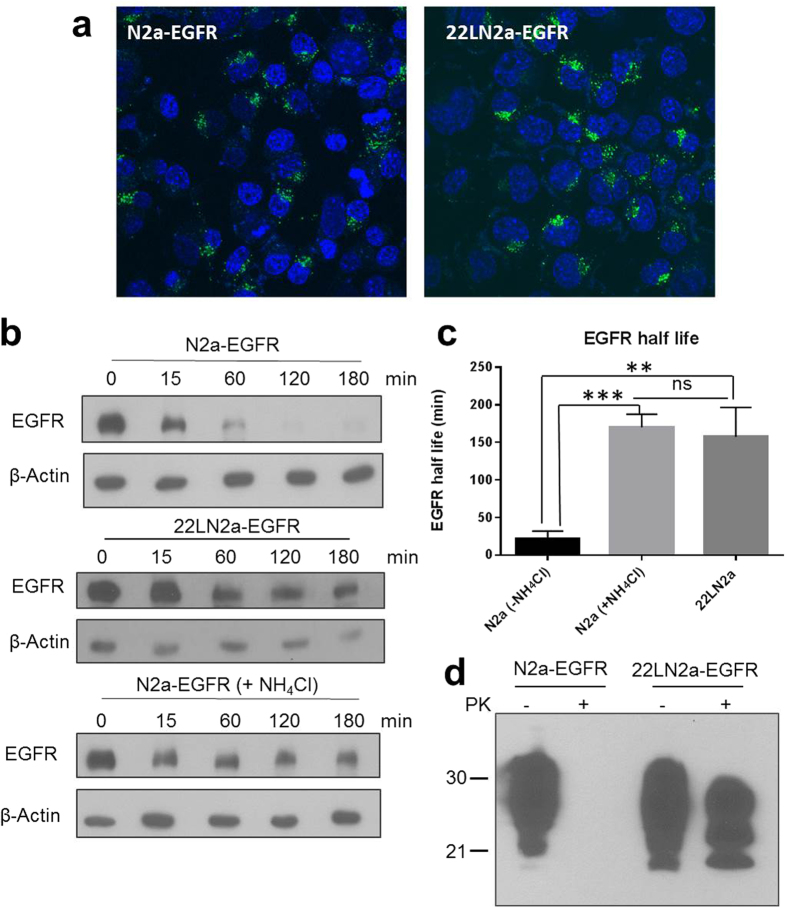
Lysosomal degradation is impaired in 22LN2a cells. (**a**) N2a and 22LN2a cells were transduced with recombinant retroviruses encoding human EGFR. Expression of EGFR was confirmed by visualising the internalisation of Alexa488-labeled EGF (green) using confocal microscopy. Nuclei were stained with Hoechst 33342 (blue). (**b**) EGFR degradation was monitored in N2a-EGFR, 22LN2a-EGFR and N2a-EGFR + NH_4_Cl cells. Upon pre-treatment with cycloheximide or NH_4_Cl (if indicated) cells were either directly lysed (0 min), or EGF (50 ng/ml) was supplemented and cells were lysed at indicated time points after EGF addition. Lysates were analysed by immunoblot using an anti-EGFR antibody (upper panel) or anti-β-actin (lower panel) to control for equal loading. (**c**) The half life of EGFR (signal intensity of 50% compared to the 0 min time point) was determined from three independent experiments upon quantification of EGFR signals and normalization against β-actin signals. Statistical analysis was performed using one-way ANOVA test followed by Tukey’s test (**p-value < 0.01; ***p-value < 0,001; ns = not significant). (**d**) Representative analysis of PrP signals in lysates of N2a-EGFR and 22LN2a-EGFR with (+PK) or without (−PK) PK digestion.

In order to characterize possible functional consequences of reduced rab7 membrane association we determined lysosomal degradation efficiency in N2a and 22LN2a cells by measuring the half-life of epidermal growth factor receptor (EGFR) which is a commonly used marker protein for lysosomal degradation^43^. First, we established N2a and 22LN2a cells that stably overexpress EGFR (N2a-EGFR and 22LN2a-EGFR, respectively) by transduction with recombinant retroviruses since we were not able to detect uptake of Alexa488-labeled EGF in N2a cells (Fig. S2a). With this method, a comparable number of transduced N2a and 22LN2a cells expressed EGFR, as demonstrated by analysing the number of cells that internalized Alexa488-labeled EGF using confocal microscopy (Fig. 5a). Quantitative single cell analysis revealed that there was no statistically significant difference in the number of EGF-positive vesicles (foci) per cell as well as the intensity of fluorescence of individual foci between N2a-EGFR and 22LN2a-EGFR (Fig. S2b). To study the degradation of EGFR, N2a-EGFR and 22LN2a-EGFR cells were pre-treated with cycloheximide. Then EGFR internalisation and its lysosomal degradation were stimulated by addition of EGF. As a positive control for lysosomal inhibition, N2a-EGFR cells were in addition treated with NH_4_Cl throughout the experiment. None of these treatments was significantly toxic to the cells as demonstrated by XTT assay (Fig. S3). Cells were lysed at the time point of EGF addition (0 min) and 15, 60, 120 and 180 min after stimulation with EGF, and EGFR levels in cell lysates were tested by immunoblot (Fig. 5b). Signals were quantified and normalized using β-actin signals which served as a loading control. Whereas in N2a-EGFR cells EGFR signals were strongly reduced after 60 min (<25%), in 22LN2a and N2a-EGFR + NH_4_Cl even after 180 min a strong EGFR signal was detectable (Fig. 5b). We have determined the EGFR half life from each individual experiment, and statistical analysis revealed a significant increase in the EGFR half life in 22LN2a-EGFR and N2a-EGFR + NH_4_Cl compared to N2a-EGFR, whereas the difference between 22LN2a-EGFR and N2a-EGFR + NH_4_Cl was not significant (Fig. 5c). PK treatment of N2a-EGFR and 22LN2a-EGFR cell lysates, followed by immunoblot analysis confirmed that 22LN2a-EGFR cells harbored high amounts of PrP^Sc^ at the time of EGFR degradation analysis (Fig. 5d).

**Figure 6:**
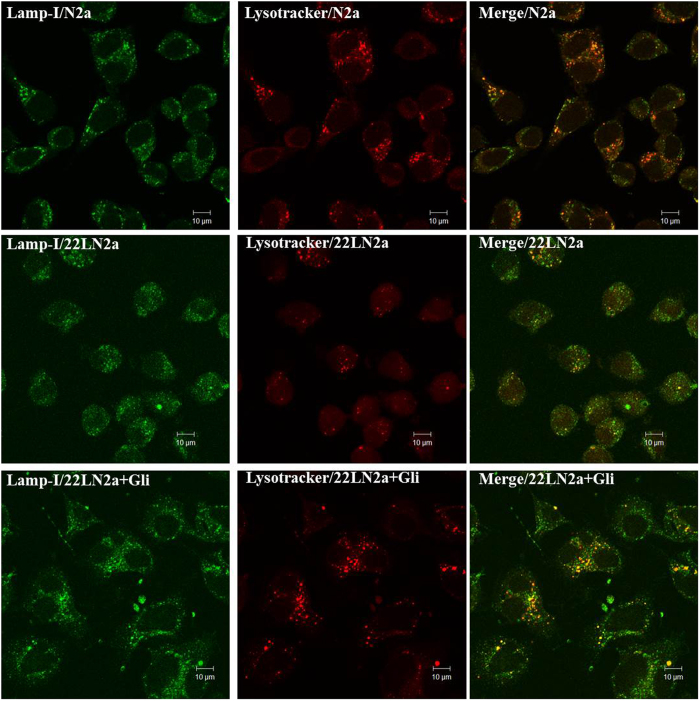
Distribution of late endosomes and lysosomes in N2a and 22LN2a cells. N2a (upper panel), 22LN2a (middle panel) and 22LNa + Gli (lower panel) were incubated with lysotracker-red (red) for 30 min. Then cells were fixed and stained using an anti-lamp-1primary antibody followed by cy-2-conjugated secondary antibody (green). Yellow colour indicates co-localisation. Confocal images were taken using a Zeiss LSM710.

If lysosomal maturation is affected by the lack of membrane-bound rab7 we expected that the numbers of lysosomes will be reduced in 22LN2a cells. To verify this, we co-stained N2a cells, 22LN2a cells and 22LN2a + Gli cells that were cured from prion infection with anti-lamp1 for detection of late endosomes and lysosomes, and with lysotracker red-dnd99 which accumulates in a pH-dependent manner in lysosomes (Fig. 6). Whereas in all three cell lines the staining with anti-lamp1 revealed a similar distribution and number of vesicles, in 22LN2a the number of vesicles accumulating lysotracker dye appeared to be considerably reduced in most of the cells. Of note, in 22LN2a + Gli cells, the pattern was similar to that in N2a cells, containing more lysotracker-positive vesicles than untreated 22LN2a cells. These data confirm that prion infection interferes with lysosomal maturation and are consistent with our finding that lysosomal degradation is reduced in prion infected cells.

**Figure 7:**
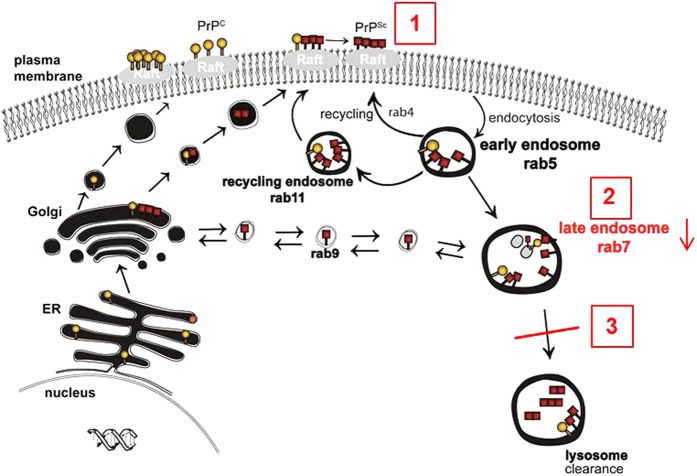
PrP^Sc^ accumulation reduces rab7 membrane attachment and lysosomal maturation. In prion infected neuronal cells, PrP^Sc^ accumulates at the plasma membrane and along the endocytic pathway (**1**). This results in an impairment of rab7 attachment to membranes (**2**). As a consequence, lysosomal maturation and overall degradation capacity are impaired (**3**).

Our findings are summarized in Fig. 7 and demonstrate that prion infection reduces the amounts of membrane-associated rab7 in neuronal cells which can be reversed by curing the cells from prion infection. Rab7 is critical for maturation of lysosomes, and consequently, the efficiency of lysosomal maturation is reduced. Since PrP^Sc^ can, albeit with a long half-life, be degraded in lysosomes, the impairment of lysosomal degradation may contribute to sustained prion propagation in persistently infected cells.

## Discussion

We demonstrate here that in two neuronal cell lines, CAD5 and N2a, infected with one or both prion strains 22L and RML the levels of membrane-bound rab7 are reduced, whereas the level of total rab7 in 22LN2a cells is comparable to that in non-infected N2a cells. Curing the cells from prion infection with two different anti-prion drugs can restore the amounts of membrane-bound rab7 to the levels found in non-infected CAD5 or N2a cells. None of the drugs exerted effects on the levels of any of the tested membrane-bound rab proteins in non-infected cells. This indicates that the decreased levels of membrane-bound rab7 are induced by prion infection. The reduction of membrane-bound rab7 is not due to an increased sensitivity to rabGDI extraction.

A number of valuable studies has been performed that aimed to identify the subcellular compartment of prion conversion^17^,^18^,^44^,^45^. In these studies, trafficking pathways were modulated by overexpression of wild type, constitutively active or transdominant negative rab proteins, as well as knockdown of endogenous rab protein expression. Thereby, it has been shown that the stimulation of retrograde transport towards the ER can enhance PrP^Sc^ formation^44^. By perturbing trafficking in the endocytic pathway, a role of early endosomes in the conversion process could be excluded and recycling endosomes^17^ as well as multivesicular bodies^18^ have been favoured as major compartments of prion conversion. Our own results demonstrate that increasing the transport from late endosomes to the trans-Golgi network by rab9 overexpression interferes with PrP^Sc^ accumulation^45^, which also points at a role of late endosomes for prion conversion.

In contrast, the aim of our current study was to investigate prion-host cell interactions, specifically how prion accumulation affects subcellular vesicle trafficking. Proteomics studies have revealed increased expression levels of RabGDI alpha upon expression of mutant PrP resulting in reduced membrane association of rab11^25^. *In vivo* studies demonstrated defects in rab7-mediated retrograde axonal transport in motor neurons of prion infected mice^24^. Both reports indicate a reduction of rab protein activity, and our data showing reduced membrane association of rab7 are in line with the latter *in vivo* study^24^. Rab7 is critical for early to late endosome maturation^46^ and for lysosomal maturation^41^. Recently, knock-down of rab7 has been shown to reduce PrP^Sc^ signals which was ascribed to an inhibition of early-to-late endosome maturation^18^. This appears contradictory to our hypothesis that reduced active rab7 levels are beneficial for prion propagation. However, our analysis by confocal microscopy revealed that lamp-1staining which detects late endosomes and lysosomes is similar in infected and non-infected cells. Furthermore, the accumulation of lysotracker dye which is pH-dependent is reduced in 22LN2a cells, indicative of a lack of lysosomes. Therefore, we argue that the acidification and transition of late endosomes into lysosomes is impaired, rather than early-to-late endosome transition.

Lysosomes are critical for protein degradation in the endocytic pathway, and as a consequence of the described alterations, we observed that the overall lysosomal degradation capacity is impaired upon prion infection. PrP^Sc^ can be degraded to a certain extent by lysosomes at a steady state^47^ and degradation can be enhanced upon activation of macroautophagy^30^,^48^,^49^. It is tempting to speculate whether prions have evolved a mechanism of escaping lysosomal degradation upon prevention of rab7 recruitment. This will ensure that the kinetics of degradation and propagation favour propagation, which is important to sustain persistent infection in neuronal cells^50^. In addition to the perturbation of PrP^Sc^ degradation, the retention time of PrP^Sc^ in late endosomes/multivesicular bodies which are major sites of prion conversion^18^ is increased if lysosomal delivery is delayed. This can further benefit prion propagation.

Evasion of lysosomal degradation is a survival mechanism that is used by bacteria or viruses, e.g. *Escherichia coli* K1^51^ which has mechanisms to prevent lysosome fusion, or hepatitis B virus X protein which interferes with lysosomal acidification^52^. The effect of *E. coli* K1 on lysosomal fusion depends on the presence of long chains of polysialic acid^51^. Interestingly, PrP^c^ and PrP^Sc^ are sialoglycoproteins^53^. Clustering of sialylated GPIs as induced upon PrP^Sc^ aggregation has been linked to neurodegeneration^54^, and PrP^c^ sialylation also controls PrP^Sc^ amplification rate and biochemical properties^55^. The observation that lysosomal maturation is reduced in prion infected cells might also be related to sialylation of PrP and a subsequent inhibition of late endosome/lysosome fusion. Interestingly, a recently published study demonstrated that expression of mutated forms of PrP can impair the function of mahogunin, a protein which is involved in amphisome/multivesicular body and lysosome fusion^56^. This functional impairment resulted in a reduced lysosomal degradation capacity^56^, and according to our results, accumulation of PrP^Sc^ has similar effects on lysosomal maturation.

What might interfere with rab7 recruitment to membranes in prion infected cells? A huge body of evidence provided by our group and others both in *in vitro* and *in vivo* systems indicates an impact of prion infection on cholesterol metabolism and membrane properties. Cholesterol synthesis is increased both in cultured neurons^32^,^33^,^35^ and in prion infected mice at preclinical stages of disease^34^, resulting in high levels of unesterified cholesterol. Unesterified cholesterol appears to be sequestered in cell membranes^35^. Furthermore, membranes of prion-infected N2a cells are more rigid^57^, which could be due to high cholesterol levels. Notably, membrane properties can affect the motility of vesicles through rab proteins^37^,^38^,^58^. Taken into account the findings about alterations in cholesterol metabolism and membrane properties in prion infection, it is likely that these alterations are the reason for reduction of rab7 membrane recruitment.

In summary, our study sheds new light into prion-host cell interactions, and points at a prion-induced mechanism that prevents PrP^Sc^ clearance. This can contribute to sustaining persistent prion replication. In addition, continuous blocking of endo-lysosomal vesicle trafficking can result in damage of neurons. Our finding that prions can modulate pathways that are non-beneficial for their propagation expands the range of information that can be encoded by protein conformation. Based on our data, we suggest modulation of vesicle trafficking as a target for treatment of prion diseases.

## Methods

### Reagents

Proteinase K and Pefabloc proteinase inhibitor were obtained from VWR. Immunoblotting was done using the enhanced chemiluminescence blotting technique (ECL plus) from Pierce. The monoclonal anti-PrP antibody (mAb) 4H11 has been described^30^. Polyclonal anti-rab7, rab9 and rab11 antibodies were purchased from Santa Cruz, Alexa488-labelled EGF and lysotracker red dnd-99 were obtained from Molecular Probes. Polyclonal antibodies against EGFR and TRAPα, respectively, were from Abcam. All other chemicals were from Sigma.

### Cell culture and treatment of cells

The cell lines N2a (ATCC-CCL131; murine brain-derived; neuroblastoma) and 22LN2a have been described^32^,^59^. The CNS cell line CAD5 has been derived from catecholaminergic neuronal cells and can be infected with multiple prion strains^60^,^61^. Cells were kept in Opti-MEM (Gibco) containing 10% fetal bovine serum (PAA laboratories) and penicillin/streptomycin (Gibco). 22LN2a cells were cured from prion infection by treatment with glivec^30^ (10 μM) or pentosan polysulfate^29^ (PPS; 1 μg/ml) for 10 days and then passaged four times before they were used for further experiments. Non-infected cells were treated in parallel. For analysis of total rab protein, cell pellets were resuspended in PBS and sonicated. Appropriate volumes of sample buffer for SDS-PAGE were added and aliquots were analysed by immunoblot.

### Cell lysis, PK digestion and immunoblot analysis

Cell lysis and immunoblot was performed as described previously^13^. For PK digestion, 50% of the cell lysate was incubated with PK (20 μg/ml) for 30 min at 37 °C. Digestion was stopped by addition of Pefabloc protease inhibitor (VWR), and samples were processed for immunoblot.

### Preparation of crude membrane extracts

The procedure has been described previously^37^. In brief, cells were swollen in Hepes buffer (10 mM, pH 7,4) for 10 min on ice, scraped into homogenization buffer (Hepes pH 7,4 20 mM, sucrose 250 mM, EDTA 1 mM, DTT 1 mM) and homogenized by passing through a 22 gauge needle. Upon centrifugation (3,000 × g, 5 min, 4 °C) membranes fractions were pelleted from postnuclear supernatants (100,000 × g, 15 min, 4 °C). Pellet (membrane) fractions were resuspended in homogenization buffer and protein concentrations were determined. For immunoblot analysis, 100 μg of protein was loaded. Signals were quantified using Image Quant TL (GE Healthcare). Statistical evaluation of results from 3 independent experiments was done using one-way ANOVA and post-hoc analysis with Tukey’s test (GraphPad Prism software).

### Rab-GDI extraction of rab proteins

RabGDI cloned into pRSET (kindly provided by Dr. Oliver Ullrich, Hamburg, Germany) was expressed in *E. coli* strain BL21 and purified as described^62^. Membrane fractions containing equal amounts of the respective rab protein were incubated in rabGDI extraction buffer (Hepes pH7.4 20 mM, KCl 100 mM, MgCl_2_ 1 mM, GDP 1 mM, BSA 0.5 mg/ml, Pefabloc) containing 0 or 3 μM of purified rabGDI for 1 h at 37 °C. Insoluble (membrane bound) proteins were separated by centrifugation (100,000 × g, 10 min, 4 °C) from solubilised rab proteins^37^. Signals were quantified using Image Quant TL (GE Healthcare). Statistical evaluation of results from at least 3 independent experiments was done using one-way ANOVA and post-hoc analysis with Tukey’s test (GraphPad Prism software).

### Retroviral transduction

VSV-glycoprotein pseudotyped retroviruses were produced using the pVPack system (Stratagene) and pBABE-EGFR (Addgene) co-transfected into HEK 293FT cells. Forty-eight hours post transfection cell culture supernatants were collected and cell debris was removed by centrifugation (120 × g, 5 min). Transduction of N2a and 22LN2a cells was performed as described^63^. Expression of EGFR was confirmed by monitoring the uptake of Alexa488-labelled EGF.

### EGFR degradation assay

N2a-EGFR and 22LN2a-EGFR were pre-treated with cycloheximide (25 μg/ml) for 90 min before stimulation with EGF (50 ng/ml). Cells were lysed after cycloheximide treatment (0 min) and 15, 60, 120 and 180 min after EGF addition. To inhibit lysosomal degradation, NH_4_Cl (10 mM) was added one hour before and during cycloheximide treatment and EGF stimulation. Equal amounts of protein were analysed by immunoblot for EGFR expression. Signals were quantified using Image Quant TL (GE Healthcare) or Quantity one software (Biorad) and normalized using β-actin signals. Statistical evaluation of results from 3 independent experiments was done using one-way ANOVA and post-hoc analysis with Tukey’s test (GraphPad Prism software).

### Immunofluorescence staining and confocal microscopy

Cells were fixed with paraformaldehyde (4%) prior to treatment with NH_4_Cl/glycine (50 mM/20 mM), triton-X 100 (0, 2%), and gelatine (0, 2%) for 10 min each. Primary antibody recognizing lamp-1 (BD Bioscience) and cy-2 conjugated secondary antibody were incubated for one hour each at room temperature. For lysosomal staining, lysotracker red dnd-99 (50 nM) was added for 30 min to the cell culture medium prior to fixation. Images were acquired using a Zeiss LSM710 confocal microscope.

## Additional Information

**How to cite this article**: Shim, S. Y. *et al.* Prion infection impairs lysosomal degradation capacity by interfering with rab7 membrane attachment in neuronal cells. *Sci. Rep.* **6**, 21658; doi: 10.1038/srep21658 (2016).

## Supplementary Material

Supplementary Information

## References

[b1] Prusiner S. B. Novel proteinaceous infectious particles cause scrapie. Science 216, 136–144 (1982). 6801762 10.1126/science.6801762

[b2] Prusiner S. B. Prions. Proc. Natl. Acad. Sci. USA 95, 13363–13383 (1998). 9811807 10.1073/pnas.95.23.13363 PMC33918

[b3] Lansbury P. T. Jr. & Caughey B. The chemistry of scrapie infection: implications of the ‘ice 9’ metaphor. Chem. Biol. 2, 1–5 (1995). 9383397 10.1016/1074-5521(95)90074-8

[b4] Caughey B., Kocisko D. A., Raymond G. J. & Lansbury, P. T., Jr. Aggregates of scrapie-associated prion protein induce the cell-free conversion of protease-sensitive prion protein to the protease-resistant state. Chem. Biol. 2, 807–817 (1995). 8807814 10.1016/1074-5521(95)90087-x

[b5] Aguzzi A. & Weissmann C. Prion diseases. Haemophilia. 4, 619–627 (1998). 9873804 10.1046/j.1365-2516.1998.440619.x

[b6] Wadsworth J. D. & Collinge J. Update on human prion disease. Biochim. Biophys. Acta 1772, 598–609 (2007). 17408929 10.1016/j.bbadis.2007.02.010

[b7] Watts J. C., Balachandran A. & Westaway D. The expanding universe of prion diseases. PLoS. Pathog. 2, e26 (2006). 16609731 10.1371/journal.ppat.0020026 PMC1434791

[b8] Stahl N., Borchelt D. R., Hsiao K. & Prusiner S. B. Scrapie prion protein contains a phosphatidylinositol glycolipid. Cell 51, 229–240 (1987). 2444340 10.1016/0092-8674(87)90150-4

[b9] Bueler H. *et al.* Mice devoid of PrP are resistant to scrapie. Cell 73, 1339–1347 (1993). 8100741 10.1016/0092-8674(93)90360-3

[b10] Gilch S. *et al.* Recognition of lumenal prion protein aggregates by post-ER quality control mechanisms is mediated by the preoctarepeat region of PrP. Traffic. 5, 300–313 (2004). 15030571 10.1111/j.1600-0854.2004.0175.x

[b11] Borchelt D. R., Scott M., Taraboulos A., Stahl N. & Prusiner S. B. Scrapie and cellular prion proteins differ in their kinetics of synthesis and topology in cultured cells. J. Cell Biol. 110, 743–752 (1990). 1968466 10.1083/jcb.110.3.743 PMC2116048

[b12] Taraboulos A., Scott M., Semenov A., Avrahami D. & Prusiner S. B. Biosynthesis of the prion proteins in scrapie-infected cells in culture. Braz. J. Med. Biol. Res. 27, 303–307 (1994). 8081243

[b13] Gilch S. *et al.* Intracellular re-routing of prion protein prevents propagation of PrP(Sc) and delays onset of prion disease. EMBO J. 20, 3957–3966 (2001). 11483499 10.1093/emboj/20.15.3957 PMC149175

[b14] Veith N. M., Plattner H., Stuermer C. A., Schulz-Schaeffer W. J. & Burkle A. Immunolocalisation of PrPSc in scrapie-infected N2a mouse neuroblastoma cells by light and electron microscopy. Eur. J. Cell Biol. 88, 45–63 (2009). 18834644 10.1016/j.ejcb.2008.08.001

[b15] Godsave S. F. *et al.* Cryo-immunogold electron microscopy for prions: toward identification of a conversion site. J. Neurosci. 28, 12489–12499 (2008). 19020041 10.1523/JNEUROSCI.4474-08.2008 PMC2796247

[b16] Borchelt D. R., Taraboulos A. & Prusiner S. B. Evidence for synthesis of scrapie prion proteins in the endocytic pathway. J. Biol. Chem. 267, 16188–16199 (1992). 1353761

[b17] Marijanovic Z., Caputo A., Campana V. & Zurzolo C. Identification of an intracellular site of prion conversion. PLoS. Pathog. 5, e1000426 (2009). 19424437 10.1371/journal.ppat.1000426 PMC2673690

[b18] Yim Y. I. *et al.* The multivesicular body is the major internal site of prion conversion. J. Cell Sci. 128, 1434–1443 (2015). 25663703 10.1242/jcs.165472 PMC4379730

[b19] Goold R. *et al.* Rapid cell-surface prion protein conversion revealed using a novel cell system. Nat. Commun. 2, 281 (2011). 21505437 10.1038/ncomms1282 PMC3104518

[b20] Magalhaes A. C. *et al.* Uptake and neuritic transport of scrapie prion protein coincident with infection of neuronal cells. J. Neurosci. 25, 5207–5216 (2005). 15917460 10.1523/JNEUROSCI.0653-05.2005 PMC6724812

[b21] Uchiyama K. *et al.* Prions disturb post-Golgi trafficking of membrane proteins. Nat. Commun. 4, 1846 (2013). 23673631 10.1038/ncomms2873

[b22] Pfeffer S. R. Rab GTPases: master regulators of membrane trafficking. Curr. Opin. Cell Biol. 6, 522–526 (1994). 7986528 10.1016/0955-0674(94)90071-x

[b23] Stenmark H. Rab GTPases as coordinators of vesicle traffic. Nat. Rev. Mol. Cell Biol. 10, 513–525 (2009). 19603039 10.1038/nrm2728

[b24] Ermolayev V. *et al.* Impaired axonal transport in motor neurons correlates with clinical prion disease. PLoS. Pathog. 5, e1000558 (2009). 19696919 10.1371/journal.ppat.1000558 PMC2723930

[b25] Massignan T. *et al.* Mutant prion protein expression is associated with an alteration of the Rab GDP dissociation inhibitor alpha (GDI)/Rab11 pathway. Mol. Cell Proteomics. 9, 611–622 (2010). 19996123 10.1074/mcp.M900271-MCP200 PMC2860234

[b26] Kovacs G. G. *et al.* Involvement of the endosomal-lysosomal system correlates with regional pathology in Creutzfeldt-Jakob disease. J. Neuropathol. Exp. Neurol. 66, 628–636 (2007). 17620988 10.1097/nen.0b013e318093ecc7

[b27] Zafar S. *et al.* Proteomics approach to identify the interacting partners of cellular prion protein and characterization of Rab7a interaction in neuronal cells. J. Proteome. Res. 10, 3123–3135 (2011). 21604690 10.1021/pr2001989

[b28] Rouvinski A. *et al.* Live imaging of prions reveals nascent PrPSc in cell-surface, raft-associated amyloid strings and webs. J. Cell Biol. 204, 423–441 (2014). 24493590 10.1083/jcb.201308028 PMC3912534

[b29] Caughey B. & Raymond G. J. Sulfated polyanion inhibition of scrapie-associated PrP accumulation in cultured cells. J. Virol. 67, 643–650 (1993). 7678300 10.1128/jvi.67.2.643-650.1993 PMC237415

[b30] Ertmer A. *et al.* The tyrosine kinase inhibitor STI571 induces cellular clearance of PrPSc in prion-infected cells. J. Biol. Chem. 279, 41918–41927 (2004). 15247213 10.1074/jbc.M405652200

[b31] Ertmer A. *et al.* The anticancer drug imatinib induces cellular autophagy. Leukemia 21, 936–942 (2007). 17330103 10.1038/sj.leu.2404606

[b32] Bach C. *et al.* Prion-induced activation of cholesterogenic gene expression by Srebp2 in neuronal cells. J. Biol. Chem. 284, 31260–31269 (2009). 19748890 10.1074/jbc.M109.004382 PMC2781524

[b33] Cui H. L. *et al.* Prion infection impairs cholesterol metabolism in neuronal cells. J. Biol. Chem. 289, 789–802 (2014). 24280226 10.1074/jbc.M113.535807 PMC3887205

[b34] Brown A. R. *et al.* Gene expression profiling of the preclinical scrapie-infected hippocampus. Biochem. Biophys. Res. Commun. 334, 86–95 (2005). 15992767 10.1016/j.bbrc.2005.06.060

[b35] Bate C., Tayebi M. & Williams A. Sequestration of free cholesterol in cell membranes by prions correlates with cytoplasmic phospholipase A2 activation. BMC. Biol. 6, 8 (2008). 18269734 10.1186/1741-7007-6-8 PMC2270799

[b36] Mukherjee S., Zha X., Tabas I. & Maxfield F. R. Cholesterol distribution in living cells: fluorescence imaging using dehydroergosterol as a fluorescent cholesterol analog. Biophys. J. 75, 1915–1925 (1998). 9746532 10.1016/S0006-3495(98)77632-5 PMC1299862

[b37] Ganley I. G. & Pfeffer S. R. Cholesterol accumulation sequesters Rab9 and disrupts late endosome function in NPC1-deficient cells. J. Biol. Chem. 281, 17890–17899 (2006). 16644737 10.1074/jbc.M601679200 PMC3650718

[b38] Chen H., Yang J., Low P. S. & Cheng J. X. Cholesterol level regulates endosome motility via Rab proteins. Biophys. J. 94, 1508–1520 (2008). 17981910 10.1529/biophysj.106.099366 PMC2212687

[b39] Takahashi M. *et al.* Cholesterol controls lipid endocytosis through Rab11. Mol. Biol. Cell 18, 2667–2677 (2007). 17475773 10.1091/mbc.E06-10-0924 PMC1924824

[b40] Ullrich O. *et al.* Rab GDP dissociation inhibitor as a general regulator for the membrane association of rab proteins. J. Biol. Chem. 268, 18143–18150 (1993). 8349690

[b41] Bucci C., Thomsen P., Nicoziani P., McCarthy J. & van D. B. Rab7: a key to lysosome biogenesis. Mol. Biol. Cell 11, 467–480 (2000). 10679007 10.1091/mbc.11.2.467 PMC14786

[b42] Vanlandingham P. A. & Ceresa B. P. Rab7 regulates late endocytic trafficking downstream of multivesicular body biogenesis and cargo sequestration. J. Biol. Chem. 284, 12110–12124 (2009). 19265192 10.1074/jbc.M809277200 PMC2673280

[b43] Ceresa B. P. & Bahr S. J. rab7 activity affects epidermal growth factor:epidermal growth factor receptor degradation by regulating endocytic trafficking from the late endosome. J. Biol. Chem. 281, 1099–1106 (2006). 16282324 10.1074/jbc.M504175200

[b44] Beranger F., Mange A., Goud B. & Lehmann S. Stimulation of PrP(C) retrograde transport toward the endoplasmic reticulum increases accumulation of PrP(Sc) in prion-infected cells. J. Biol. Chem. 277, 38972–38977 (2002). 12163492 10.1074/jbc.M205110200

[b45] Gilch S., Bach C., Lutzny G., Vorberg I. & Schatzl H. M. Inhibition of cholesterol recycling impairs cellular PrP(Sc) propagation. Cell Mol. Life Sci. 66, 3979–3991 (2009). 19823766 10.1007/s00018-009-0158-4 PMC2777232

[b46] Girard E. *et al.* Rab7 is functionally required for selective cargo sorting at the early endosome. Traffic. 15, 309–326 (2014). 24329906 10.1111/tra.12143

[b47] Luhr K. M., Nordstrom E. K., Low P. & Kristensson K. Cathepsin B and L are involved in degradation of prions in GT1-1 neuronal cells. Neuroreport 15, 1663–1667 (2004). 15232303 10.1097/01.wnr.0000134931.81690.34

[b48] Aguib Y. *et al.* Autophagy induction by trehalose counteracts cellular prion infection. Autophagy. 5, 361–369 (2009). 19182537 10.4161/auto.5.3.7662

[b49] Heiseke A., Aguib Y., Riemer C., Baier M. & Schatzl H. M. Lithium induces clearance of protease resistant prion protein in prion-infected cells by induction of autophagy. J. Neurochem. 109, 25–34 (2009). 19183256 10.1111/j.1471-4159.2009.05906.x

[b50] Ghaemmaghami S. *et al.* Cell division modulates prion accumulation in cultured cells. Proc. Natl. Acad. Sci. USA 104, 17971–17976 (2007). 17989223 10.1073/pnas.0708372104 PMC2084281

[b51] Kim K. J., Elliott S. J., Di C. F., Stins M. F. & Kim K. S. The K1 capsule modulates trafficking of E. coli-containing vacuoles and enhances intracellular bacterial survival in human brain microvascular endothelial cells. Cell Microbiol. 5, 245–252 (2003). 12675682 10.1046/j.1462-5822.2003.t01-1-00271.x

[b52] Liu B. *et al.* Hepatitis B virus X protein inhibits autophagic degradation by impairing lysosomal maturation. Autophagy. 10, 416–430 (2014). 24401568 10.4161/auto.27286 PMC4077881

[b53] Stahl N. *et al.* Glycosylinositol phospholipid anchors of the scrapie and cellular prion proteins contain sialic acid. Biochemistry 31, 5043–5053 (1992). 1350920 10.1021/bi00136a600

[b54] Bate C. & Williams A. Clustering of sialylated glycosylphosphatidylinositol anchors mediates PrP-induced activation of cytoplasmic phospholipase A 2 and synapse damage. Prion. 6, 350–353 (2012). 22895089 10.4161/pri.21751 PMC3609062

[b55] Katorcha E., Makarava N., Savtchenko R., D’Azzo A. & Baskakov I. V. Sialylation of prion protein controls the rate of prion amplification, the cross-species barrier, the ratio of PrPSc glycoform and prion infectivity. PLoS. Pathog. 10, e1004366 (2014). 25211026 10.1371/journal.ppat.1004366 PMC4161476

[b56] Majumder P. & Chakrabarti O. Mahogunin regulates fusion between amphisomes/MVBs and lysosomes via ubiquitination of TSG101. Cell Death. Dis. 6, e1970 (2015). 26539917 10.1038/cddis.2015.257 PMC4670916

[b57] Wong K. *et al.* Decreased receptor-mediated calcium response in prion-infected cells correlates with decreased membrane fluidity and IP3 release. Neurology 47, 741–750 (1996). 8797473 10.1212/wnl.47.3.741

[b58] Lebrand C. *et al.* Late endosome motility depends on lipids via the small GTPase Rab7. EMBO J. 21, 1289–1300 (2002). 11889035 10.1093/emboj/21.6.1289 PMC125356

[b59] Grassmann A., Wolf H., Hofmann J., Graham J. & Vorberg I. Cellular aspects of prion replication *in vitro*. Viruses. 5, 374–405 (2013). 23340381 10.3390/v5010374 PMC3564126

[b60] Mahal S. P. *et al.* Prion strain discrimination in cell culture: the cell panel assay. Proc. Natl. Acad. Sci. USA 104, 20908–20913 (2007). 18077360 10.1073/pnas.0710054104 PMC2409240

[b61] Qi Y., Wang J. K., McMillian M. & Chikaraishi D. M. Characterization of a CNS cell line, CAD, in which morphological differentiation is initiated by serum deprivation. J. Neurosci. 17, 1217–1225 (1997). 9006967 10.1523/JNEUROSCI.17-04-01217.1997 PMC6793738

[b62] Liu P. *et al.* Rab-regulated interaction of early endosomes with lipid droplets. Biochim. Biophys. Acta 1773, 784–793 (2007). 17395284 10.1016/j.bbamcr.2007.02.004 PMC2676670

[b63] Maas E. *et al.* Scrapie infection of prion protein-deficient cell line upon ectopic expression of mutant prion proteins. J. Biol. Chem. 282, 18702–18710 (2007). 17468101 10.1074/jbc.M701309200

