# Supplementary material for: Prion infection impairs lysosomal degradation capacity by interfering with rab7 membrane attachment in neuronal cells
Source: Sci Rep. 2016 Feb 11;6:21658. doi: 10.1038/srep21658 (PMC4749993; doi:10.1038/srep21658)
Supplement: Supplementary Information [file srep21658-s1.pdf]

Supplementary Information

Prion infection impairs lysosomal degradation capacity by interfering with rab7 membrane attachment in neuronal cells

Su Yeon Shim[1], Srinivasarao Karri[1], Sampson Law[2], Hermann M. Schatzl[2,3], Sabine Gilch[1,3*]

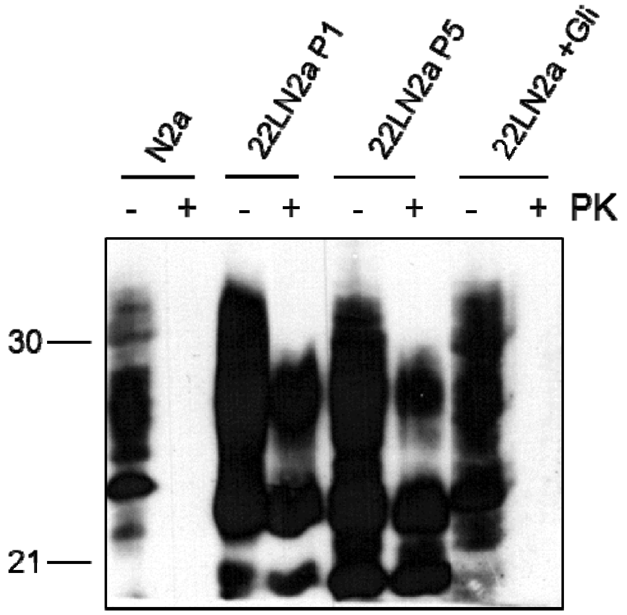

Supplementary Figure 1 Crude membrane preparations harbor PrP^{Sc} Crude membranes were prepared from N2a, 22LN2a (passage (P) 1 and 5) and 22LN2a +Gli cells and subjected to PK digestion (20 µg/ml, 30 min, 37° C; +PK) or not (-PK). Samples were analysed by immunoblot for PrP content using anti-PrP mAb 4H11. Signals in samples +PK represent PrP^{Sc}, signals in samples –PK show both PrP^{c} and PrP^{Sc}. Therefore, PrP signals in lysates of 22LN2a appear higher than those in non-infected N2a cells or 22LN2a+Gli cells which both do not accumulate PrP^{Sc}.

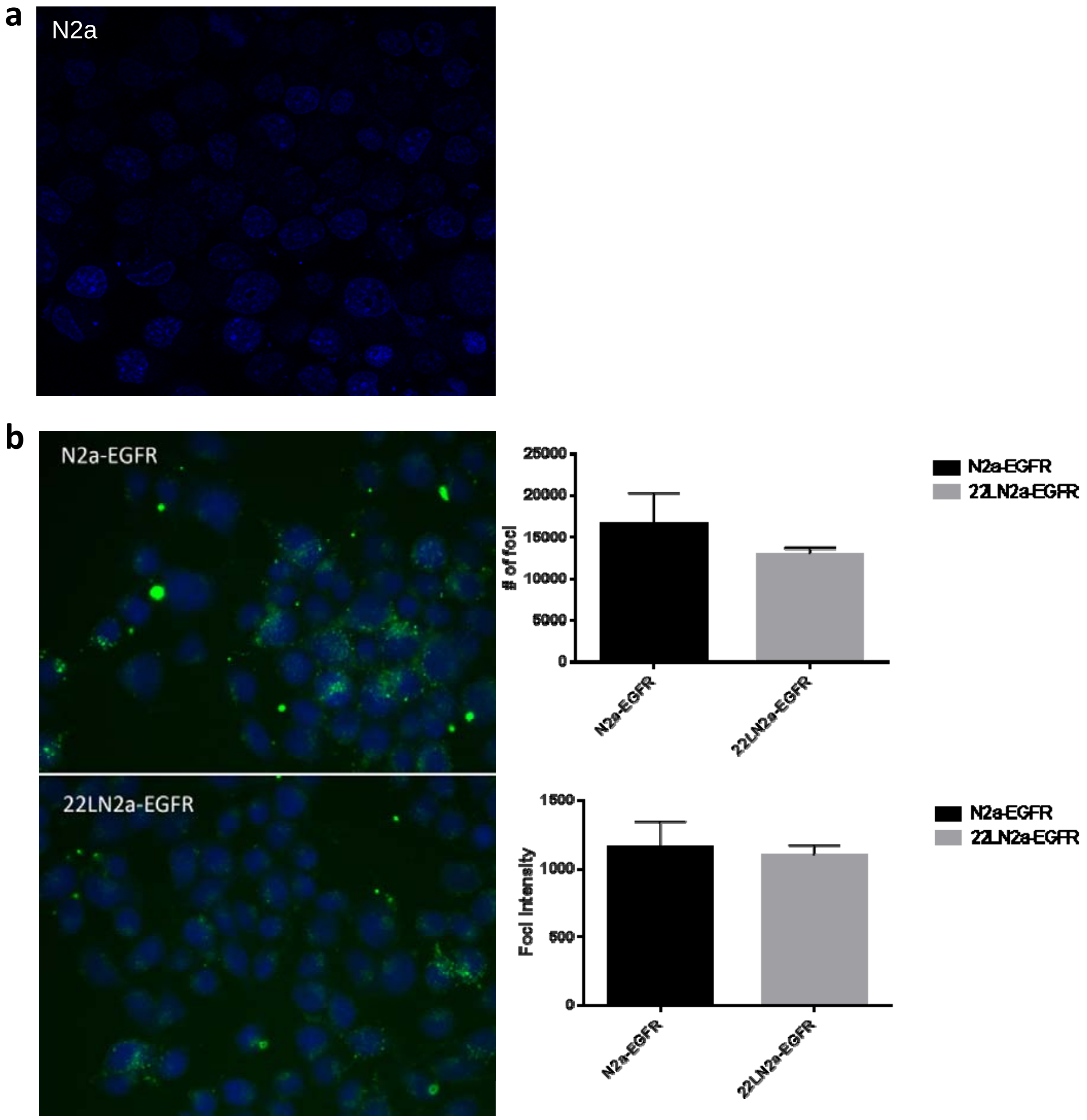

Supplementary Figure 2 No difference in internalized EGF signals between N2a-EGFR and 22LN2a-EGFR. (a) N2a cells were incubated with Alexa488-labelled EGF to test expression of EGFR. No intracellular EGF signal was visible, indicating that N2a cells do not express EGFR. Image was taken using a Zeiss LSM710 confocal microscope. **(b)** 10^5 N2a-EGFR and22LN2a-EGFR cells were seeded overnight into 24-well plates. Alexa488-labeled EGF was added to the cells for 30 min, then cells were fixed using 4% paraformaldehyde for 10 min. Cell nuclei were stained with 4',6-diamidino-2-phenylindole (DAPI; Life Technologies) in PBS. The plates were imaged using an INCell Analyzer 2000 (GE Healthcare) with a Nikon 10x/0.45 Plan Apochromat objective, and 12 fields of view were captured. Three independent wells and approximately 10,000 cells each were analysed. Images were taken with two excitation/emission filter sets (350/455 nm for DAPI and 490/525 nm for Alexa Fluor 488). Exposures were equal

for all images captured. A representative field of view for each cell line, acquired using a Nikon 40x objected is presented. Images were analyzed using the INCell Analyzer 1000 Workstation (3.7, build 1461, GE Healthcare). Cell nuclei were first identified in the DAPI channel using "top-hat" segmentation, then Granularity Analysis Module was used to evaluate the number of foci and fluorescence intensity per foci of internalized Alexa Fluor 488-EGF. Statistical evaluation was done using student's t-test (GraphPad Prism software), no significant differences were found between N2a-EGFR and 22LN2a-EGFR cells. Bars represent standard deviation.

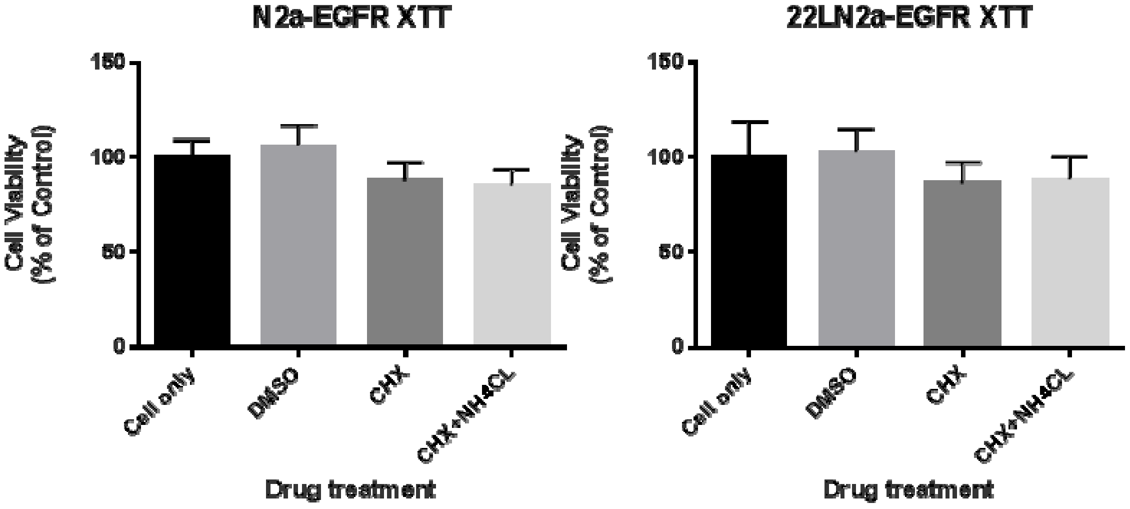

Supplementary Figure 3 No toxicity of cycloheximide and NH_4Cl treatment N2a-EGFR or 22LN2a-EGFR were seeded at a density of $5x10^4$ in 96well plates. The next day, cells were either not treated (cell only), treated with DMSO (solvent control), cycloheximide (25 µg/ml) or cycloheximide and NH_4Cl (10 mM) as described for EGFR degradation assay in the Methods section. XTT assay was performed according to the manufacturer's protocol. The absorbance was determined, and the values of untreated cells were set to 100%. All other values were expressed as percentage of the control. No significant differences were observed between treated and untreated cells (one-way ANOVA and Tukey's test; GraphPad Prism software).
